# Patterns of Harmful Alcohol Consumption among Truck Drivers: Implications for Occupational Health and Work Safety from a Systematic Review and Meta-Analysis

**DOI:** 10.3390/ijerph15061121

**Published:** 2018-05-30

**Authors:** Nicola Luigi Bragazzi, Guglielmo Dini, Alessandra Toletone, Alborz Rahmani, Alfredo Montecucco, Emanuela Massa, Alessia Manca, Ottavia Guglielmi, Sergio Garbarino, Nicoletta Debarbieri, Paolo Durando

**Affiliations:** 1 Department of Health Sciences (DISSAL), Postgraduate School of Occupational Medicine, University of Genoa, Genoa 16132, Italy; guglielmo.dini@unige.it (G.D.); alborz.rahmani@edu.unige.it (A.R.); alfredo.montecucco@edu.unige.it (A.M.); emanuela.massa@edu.unige.it (E.M.); alessia.manca@edu.unige.it (A.M.); nicoletta.debarbieri@hsanmartino.it (N.D.); 2 Occupational Medicine Unit, Policlinico San Martino Hospital, 16132 Genoa, Italy; 3 Occupational Medical Service, Local Health Unit 1, Liguria Regional Healthcare System, 18038 Imperia, Italy; a.toletone@asl1.liguria.it; 4 Department of Neuroscience, Rehabilitation, Ophthalmology, Genetics, Maternal and Child Health (DINOGMI), University of Genoa, 16132 Genoa, Italy; ottavia.guglielmi@gmail.com (O.G.); sgarbarino.neuro@gmail.com (S.G.)

**Keywords:** harmful use of alcohol, truck-drivers, occupational health and well-being, systematic review and meta-analysis

## Abstract

Alcohol consumption is one of the main causes of productivity losses arising from absenteeism, presenteeism, and workplace injuries. Among occupational categories most affected by the use of this substance, truck drivers are subject to risk factors and risky behaviors that can have a serious impact on their health, their work, and the general road safety. The use of alcohol during truck-driving activities is, indeed, an important risk factor for traffic accidents. The present systematic review and meta-analysis aims at synthesizing the literature regarding harmful alcohol consumption patterns among truck drivers in a rigorous way. A ‘binge drinking’ prevalence of 19.0%, 95% confidence interval or CI (13.1, 26.9) was present. An ‘everyday drinking’ pattern rate of 9.4%, 95% CI (7.0, 12.4) was found, while the rate of alcohol misuse according to the “Alcohol Use Disorders Identification Test” (AUDIT)—“Cut down-Annoyed-Guilty-Eye opener questionnaire” (CAGE) instruments was computed to be of 22.7%, 95% CI (14.8, 33.0). No evidence of publication bias could be found. However, there is the need to improve the quality of published research, utilizing standardized reliable instruments. The knowledge of these epidemiological data can be useful for decision makers in order to develop, design, and implement ad hoc adequate policies.

## 1. Introduction

“If you bought it, a truck brought it.” A popular saying.

Alcohol consumption is among the main causes of absenteeism, presenteeism, and workplace injuries [1,2,3]. In the USA, alcohol-induced impairment directly affects an estimated 15% of the workforce and causes more than 22% of the deaths as a result of injuries at work [4,5].

Concerning the relationship between using alcohol and accidents/injuries, studies have shown that drinking alcohol before driving was responsible for approximately 21–30% of car crash injuries in the general driving population [6,7,8]. Alcohol, indeed, impairs brake reaction time, steering responsiveness, and lane control. It also increases the tendency of speeding on the road and other high risk driving behaviors. It is noteworthy to remember that 5.9% of all global deaths result from the harmful use of alcohol, as well as 5.1% of the world’s diseases and injuries [9]. Over 1.2 million people die, and up to 50 million nonfatal injuries incur each year because of road accidents [10]. Fifteen percent of all deaths and 13% of all injuries and disabilities caused by traffic crashes are attributable to alcohol [11,12].

Truck drivers are an important part of worldwide trade and economy. In the field of transportation, trucks are used in freight movement over land, transporting raw materials, livestock, and finished goods from manufacturing plants to retail distribution centers. In the construction industry, they can be used as dump trucks and portable concrete mixers. It is estimated that there are approximately 1.7 million long-haul truck drivers in the USA [13], 260,000 in Australia [14], and more than 1.5 million in European countries [15].

Truck drivers have been reported as a highly vulnerable working population due to different risk factors [16,17,18] including hypertension, fatigue [19], obstructive sleep apnea (OSA) and sleep deprivation [20,21], and insufficient physical activity [22]. Other risk factors are exposure to diesel exhaust and risk of developing lung cancer [23], poor diet, obesity, dyslipidemia, and other metabolic disorders [24]. Furthermore, they are prone to risky behaviors and lifestyles such as smoking, drinking, using psychoactive substances, and having casual sexual contacts [25]. These risk factors and risky behaviors can have a relevant impact on their health and work ability [16,17,18], as well as work safety, increasing the risk of injuries and traffic accidents [26,27,28]. They can, indeed, lead to impairment in the physical and mental health, and together with anxiety and stressful conditions due to irregular working schedules, night shifts, the need for prolonged mental alertness, and high productivity demands [16,29,30,31], increase the rate of motor vehicle accidents (MVAs) [32,33,34]. Studies conducted over the past 20 years have shown a significant association between alcohol misuse and traffic accidents, even if the precise role it may play in the disproportionate involvement of large motor vehicles (e.g., trucks and busses) in MVAs remains equivocal [30]. Although countries have legislations that regulate the driving time in the transport sector, truck drivers still have to work for long uninterrupted shifts [35]. This could encourage the high use of stimulants, drugs, and alcohol [36].

Evidence from other occupations and work settings suggests that identifying and then intervening to alter workplace conditions associated with alcohol misuse may be an important means of prevention [37,38,39,40,41,42]. Major international organizations such as the World Health Organization (WHO) [43], the Council of the European Union [44], and the International Labor Office (ILO) [45] in several documents have maintained the need to actualize policies and programs focused on the issue of alcohol and work, pointing to the prevention of alcohol-related damage as a priority and encouraging actions to combat the alcohol use at the workplace by adopting specific measures. The alcohol consumption in truck drivers, besides being detrimental for the health, represents an important public and occupational safety concern, in that this work category is at high risk of occupational accidents and can jeopardize the safety of others. Nevertheless, in our country the extent of the problems related to the use of alcohol in the occupational category of truck-drivers is still unknown in depth, due to the paucity of available data, that can adequately inform measures to intervene at the workplace, together with shortcomings in legislation which impact on the collaboration between the stakeholders involved [46].

Therefore, the aim of this study was to fill this gap of knowledge, evaluating the prevalence rate of alcohol use and the harmful patterns of consumption among truck drivers; carrying out a systematic review and meta-analysis in such a way as to provide a scientific overview of this issue in order to equip the decision makers and the stakeholders with an updated synthesis of relevant studies.

Our study adds to the recently published work by Girotto et al. [16] in that it significantly updates and expands the published systematic review and performs a rigorous quantitative synthesis of the available scientific evidences and systematically studies the determinants of alcohol use among truck drivers.

## 2. Materials and Methods

### 2.1. Registration of Protocol with International Prospective Register of Systematic Reviews

The protocol of the present study has been reported according to the “Preferred Reporting Items for Systematic Reviews and Meta-Analysis—Protocols” (PRISMA-P) guidelines [47]. In accordance with these guidelines, the systematic review protocol has been registered with the International Prospective Register of Systematic Reviews (PROSPERO) [48] on 1 April 2016 (registration number CRD42016037077) [49].

The results of the study are reported in line with the PRISMA guidelines [50].

### 2.2. Data Sources and Search Strategy

A systematic literature search has been performed searching different scholarly databases, including nine different bibliographic thesauri (namely, PubMed/MEDLINE (NLM), Scopus, SciVerse ScienceDirect, Science Citation Index Expanded (SCIE) and Social Sciences Citation Index from ISI/Web of Science, ProQuest Research Library, ABI/INFORM, CBCA), via the UNO per TUTTI Primo Central (Ex Libris) platform databases.

All prevalence studies on alcohol use among truck drivers were included in the current study. The search was performed using the following search terms: “(truckers OR truck drivers OR lorry OR commercial vehicles OR large good vehicles OR large vehicles OR heavy vehicles OR long vehicles OR trucking industry OR haul transport) AND (alcohol OR ethanol)”. The search strategy was adapted for the other databases. Additionally, we searched reference lists of the chosen studies and prior reviews. We extensively mined different databases and we used a broad keyword string in order to capture the highest number of potentially relevant studies, minimizing the chances of missing pertinent items.

When it was not possible to make a decision on a study’s inclusion or exclusion based on the title and/or abstract, the full text of the study was examined (Table 1).

### 2.3. Study Screening and Selection

Once retrieved via the UNO per TUTTI Primo Central (Ex Libris) platform databases, duplicate studies were automatically removed. The list of non-redundant items was handled with the open source Review Manager 5 (RevMan 5) software.

The studies have been independently screened by two authors (NLB, GD) looking at study titles and/or abstracts for potential eligibility. Screening questions were developed and pilot-tested with a subset of records before implementation (Table 2). Disagreement was assessed using Cohen’s κ statistics and has been resolved through discussion; a third reviewer (AM) has been involved if necessary.

We have provided tables with characteristics of included studies and of excluded studies with reasons for their exclusion (Table 3, Table 4 and Table 5).

Studies meeting the following PICOS/PECOS criteria were considered for inclusion:

- P (population): truck-drivers.
- E (exposure): harmful use of alcohol.
- C (comparators): no comparators were considered in the present systematic review and meta-analysis.
- O (outcome/outcomes): prevalence of use of alcohol among truck drivers.
- S (study design): original studies designed as prevalence studies.
- Language: all languages available.
- Time: no time restraint.

#### 2.3.1. Alcohol Consumption Pattern

In this systematic review and meta-analysis, we followed the WHO terminology related to alcohol consumption: “health-wise” (non-hazardous), “hazardous”, and “harmful” alcohol use. The following paragraphs provide the readers with an overview of these definitions. We focused on alcohol pattern (frequency of drinking and number of drinks per occasion/event) rather than the mean alcohol intake, as the latter is an incomplete risk predictor of alcohol-related harm.

#### 2.3.2. “Harmful Alcohol Use”

The tenth edition of International Classification of Diseases (ICD-10) developed by the WHO defines “harmful alcohol use” as a pattern of substance use that causes damage to physical or mental health. This definition was closely similar to the concept of “alcohol abuse” developed by the American Psychiatric Association (APA) in the Diagnostic and Statistical Manual of Mental Disorders—Fourth edition (DSM-IV). Both of these concepts were introduced in order to gather clinically important problems associated with alcohol consumption that nonetheless could not be characterized as “alcohol dependence”.

#### 2.3.3. “Binge Drinking”

One pattern of harmful use is called “binge drinking”, defined by the National Institute on Alcohol Abuse and Alcoholism (NIAAA) as a pattern of drinking four or more drinks for women, five or more drinks for men, in a two hour timeframe, which typically brings blood alcohol concentration (BAC) levels to 0.8 g/L.

This pattern is similar to another definition called “heavy episodic drinking” (HED), defined as drinking at least 60 grams or more of pure alcohol on at least one occasion in the past 30 days. Although the two terms are often used synonymously, we decided to use the former because it is more widely used by researchers.

#### 2.3.4. “Everyday Drinking”

“Everyday drinking” among professional drivers can be considered as a pattern of consumption that increases the risk of accidents, which can therefore be harmful. Although this pattern has been referred to with several denominations, such as “daily drinking”, “continuous drinking”, and “steady drinking”, we preferred “everyday drinking” because it has been correlated to problematic use and because it is the most unambiguous term.

#### 2.3.5. “AUDIT/CAGE”

The Diagnostic and Statistical Manual of Mental Disorders - Fifth edition (DSM-V) introduced another concept, the “Alcohol Use Disorder” (AUD), which both overcomes the distinction between “abuse/harmful use” and “dependence” and includes them in a single category. The WHO has developed a tool to identify this pattern of use called Alcohol Use Disorder Identification Tool (AUDIT). A shorter three item version, called AUDIT-C identifies harmful patterns of consumption, such as frequency of drinking and “binge drinking” episodes, while the full 10-item AUDIT investigates symptoms of alcohol dependence as well. The CAGE (Cut down-Annoyed-Guilty-Eye) questionnaire is another tool previously developed to identify at risk alcohol users, an acronym for typical symptoms such as feeling the need to cut-down the amount of alcohol, being annoyed by criticism, feeling guilt, and using ethanol as an eye-opener in the morning.

#### 2.3.6. “Truck Drivers”

In this report, “truck-drivers” are defined as any person whose activities involve driving lorry or commercial vehicles with the following characteristics: large, heavy or long vehicles.

### 2.4. Appraisal of Study Quality 

Two reviewers are content experts (AM, GD) and one reviewer (NLB) is an experienced biostatistician/epidemiologist. The contents experts have only assessed potential publications with respect to the appropriateness of the research questions tested. The biostatistician has only evaluated the appropriateness of methods employed. Disagreement has been resolved by consensus.

The “Joanna Briggs Institute Critical Appraisal tools for use in JBI Systematic Reviews—Checklist for Prevalence Studies” has been used to assess the quality of studies included in the current systematic review and meta-analysis. This tool explores different domains of quality: namely, (i) the appropriateness of the sample frame to address the target population; (ii) the participants sampling technique; (iii) the adequateness of the sample size; (iv) the completeness of the description and details concerning the study subjects and the setting; (v) the coverage of the sample; (vi) the validity of the methods and (vii) their reliability; (viii) the appropriateness of the statistical analyses; and (ix) the adequateness of the response rate. 

Concerning the third domain, the adequateness of the sample size was computed using the formula:

$$n=\frac{Z^{2}\cdot P\;\left(1-P\right)}{d^{2}}$$

where *n* is the sample size, *Z* is the *Z* statistic for a given level of confidence (1.96), *P* is the expected prevalence or proportion (in proportion of one; if, for instance, 20%, *P* is 0.2), and *d* is the precision (in proportion of one; if 5%, *d* = 0.05).

Expecting a prevalence of alcohol consumption rate in the range 9–19%, an adequate sample size should comprise a minimum of 131–236 subjects.

### 2.5. Statistical Analysis 

For the meta-analysis, data have been extracted from the studies using a standardized documentation form (Table 2). Prevalence ratios were calculated as effect size (ES) estimates. The 95% confidence intervals (CIs) were also generated. More in detail, the logit transformation (*l*) approach was utilized in the current meta-analysis, being one of the possible approaches for pooling together raw prevalence data. The following equation was used to compute *l*:

$$l=ln\left(\frac{p}{1-p}\right)$$

where *p* is the prevalence proportion.

Variance was computed using the equation:

$$Var\left(l\right)=\frac{1}{N\cdot p}+\frac{1}{N\cdot \left(1-p\right)}$$

where *N* is the population size.

The pooled *l* was subsequently back-transformed to a proportion using the equation:

$$p=\frac{e^{l}}{e^{l}+1}$$

Additional analyses were performed after stratification considering all the variables listed in Table 2. Meta-analyses were carried out using the commercial software MedCalc Statistical Software version 16.8.4 (MedCalc Software bvba, Ostend, Belgium; https://www.medcalc.org; 2016) and Comprehensive Meta-Analysis CMA v3. 

### 2.6. Heterogeneity and Sensitivity Analysis 

Statistical heterogeneity has been assessed using the *I*^2^ statistic. *I*^2^ more than 50% was regarded as substantial heterogeneity [51,52]. To identify sources of variation, further stratification was performed relative to study quality and to performance of confirmatory tests. In addition, for the sensitivity analyses, the stability of the pooled estimate with respect to each study was investigated by excluding individual studies from the analysis. 

### 2.7. Publication Bias 

Potential publication bias has been extensively investigated in the current systematic review and meta-analysis. First, we have visually inspected the funnel plot, looking at asymmetry of the graph. The funnel plot chosen in the current meta-analysis is the funnel plot of precision by logit event rate. 

If asymmetry was present based on visual assessment, we performed exploratory analyses to investigate and adjust this using the Duval and Tweedie’s trim-and-fill analysis [53]. In addition, the probability of publication bias has been tested using the Egger’s linear regression test [54]. In conclusion, it should be emphasized that, in presence of statistically significant heterogeneity and with less than ten studies included, the findings of these tests should be interpreted with caution. 

## 3. Results

Seventeen studies have been included in the present systematic review and meta-analysis [8,12,26,27,55,56,57,58,59,60,61,62,63,64,65,66,67]. More in detail, from an initial list of 108,948 articles, after removing duplicates, 65,664 items remained. A pool of 65,632 articles were excluded, being deemed not relevant/pertinent with the research question. The full-text of 36 articles was assessed for eligibility, leading to 19 items excluded with reason [28,38,39,68,69,70,71,72,73,74,75,76,77,78,79,80,81,82,83]. Twelve questionnaire-based studies were excluded because they did not utilize validated, reliable instruments (such as CAGE or AUDIT), thus making the pooling of figures and their comparison methodologically unfeasible. 

Of these studies, seven articles reported an unspecified alcohol consumption pattern, whereas two studies reported a generic consumption in the last year, meaning that the truck driver had consumed any alcoholic drink during the last year. While this could be useful to identify drinkers and non-drinkers, it is not—in the authors’ opinion—useful for evaluating harmful consumption patterns, which is the main objective of the present study. Finally, one study reported alcohol consumption in terms of one per day, another study in terms of grams per kilogram, whilst one study counted the number of drinks per week. 

Seven studies conducted biological monitoring of alcohol consumption (two using breath analysis, two utilizing urine samples, two saliva samples, and one blood). 

**Figure 1:**
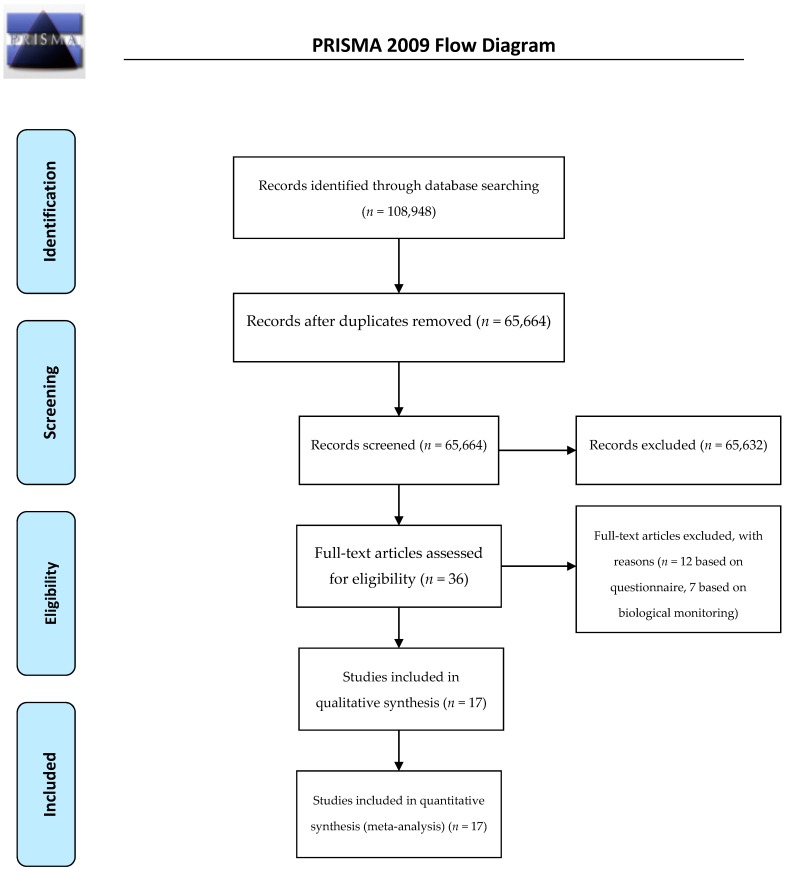
Flow-chart of the current systematic review and meta-analysis of alcohol consumption rate among truck drivers.

Seventeen studies were retained in the qualitative synthesis of the literature and included in the quantitative synthesis (meta-analysis), as pictorially shown in Figure 1.

Table 3 reports studies excluded with reason, whereas the main characteristics of studies included in the meta-analysis are described in Table 4. In Table 5, the critical appraisal of the methodological quality of the retained studies is reported.

### 3.1. Systematic Review and Meta-Analysis of Alcohol Consumption Rates among Truck Drivers

The total study population comprised 11,574 truck drivers, with sample sizes ranging from 91 to 2945 participants. Mean age ranged from 33.8 to 44.0 years old. Male percentage went from 89.0% to 100.0%. Ten studies were performed in Brazil, whereas two studies were carried out in the USA. The remaining studies were conducted in Morocco (one study), the Netherlands (one study), Italy (one study), Nigeria (one study) and Pakistan (one study). Mean work-load ranged from 11.1 to 12.7 h per day and was reported only in four studies. Night-shifters (reported in four studies) varied from 12.5% to 33.0% of participants. Schooling level was described in ten studies: primary education was achieved by 30.8–81.0% of participants, according to the study. Experience years went from 10.0 to 18.1 years (reported in eight studies). Only three studies reported the percentage of truck drivers working for companies (range 40.5–60.9%).

### 3.2. Binge Drinking among Truck Drivers

**Figure 2:**
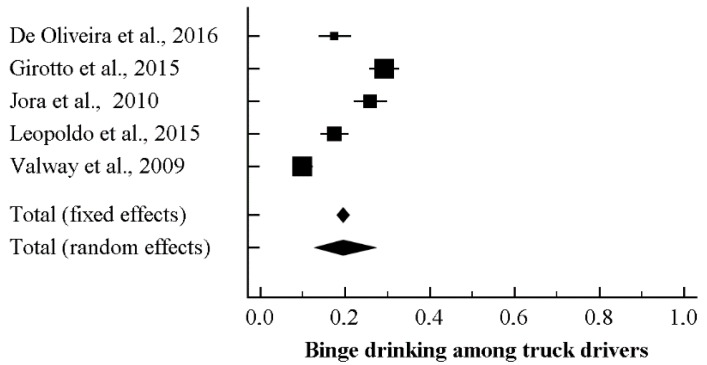
Forest plot for binge drinking among truck-drivers.

**Figure 3:**
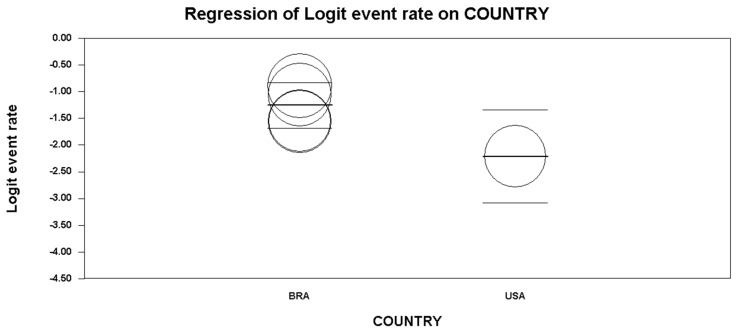
Meta-regression analysis showing statistically significant different binge drinking patterns in Brazil (BRA) and in the United States (USA), among truck-drivers.

**Figure 4:**
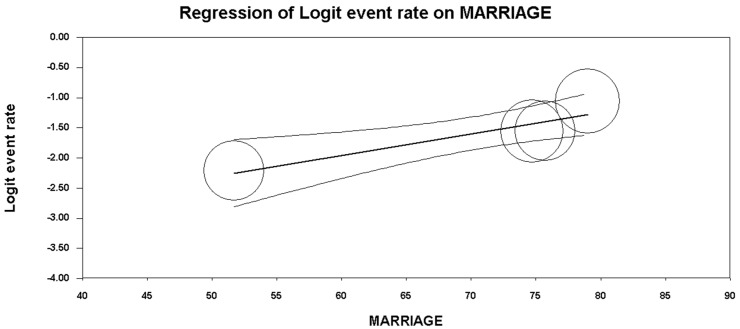
Meta-regression analysis for marriage (in percentage) among truck-drivers, showing that there is a statistically significant association between marital status and binge drinking (i.e., a higher marriage percentage correlated with a lower binge drinking pattern rate).

**Figure 5:**
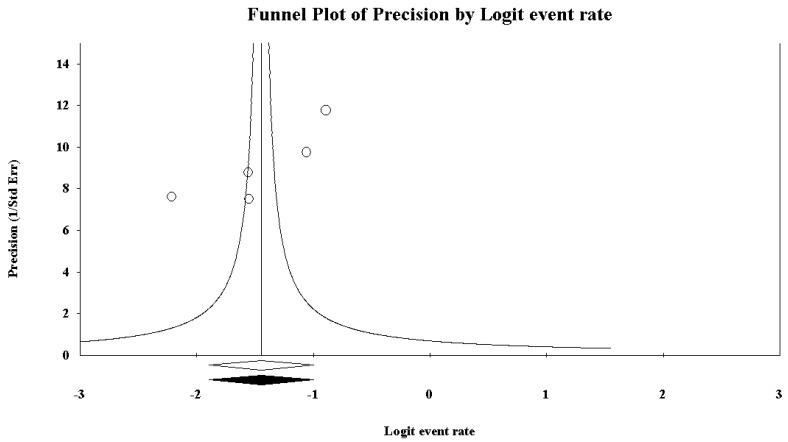
Funnel plot for binge drinking among truck-drivers, showing no evidence of publication bias.

Five studies reported data concerning binge drinking among truck drivers. Based on the *I*^2^ value (95.33), a random-effects model was performed. A rate of 19.0%, 95% CI (13.1, 26.9) was found (Figure 2, showing the forest plot). At the meta-regression analysis, country resulted a statistically significant moderator (intercept = −1.26, standard error = 0.17, 95% CI (−1.60, −0.92), *z*-value = −7.23, *p* = 0.0000, variance inflation factor or VIF = 1.24; Country = −0.96, standard error = 0.39, 95% CI (−1.73, −0.18), *z*-value = -2.42, *p* = 0.0154, VIF = 1.00) (Figure 3). Marriage was another significant moderator (intercept = −4.10, standard error = 0.75 (95% CI −5.56 to −2.63), *z*-value = −5.49, *p* = 0.0000, VIF = 44.73; marriage = 0.04, standard error = 0.01, 95% CI (0.02, 0.06), *z*-value = 3.42, *p* = 0.0006, VIF = 1.00) (Figure 4). No other statistically significant moderators could be detected. No evidence of publication bias could be found, both visually inspecting the funnel plot (Figure 5) and conducting the Duval and Tweedie’s trim-and-fill analysis, while the Egger’s linear regression test (intercept = −21.65, standard error = 6.37, 95% CI (−41.91, −1.40), *p* = 0.04241) yielded the statistical significance (Table 6).

### 3.3. “Everyday Drinking” Pattern among Truck Drivers

**Figure 6:**
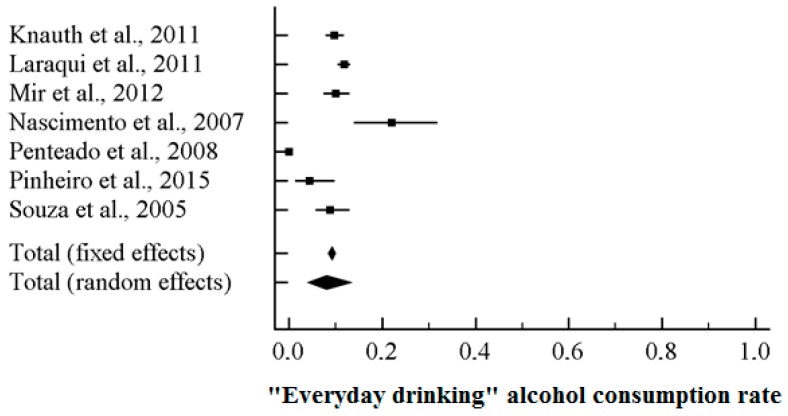
Forest plot for “everyday drinking” consumption rate among truck-drivers.

**Figure 7:**
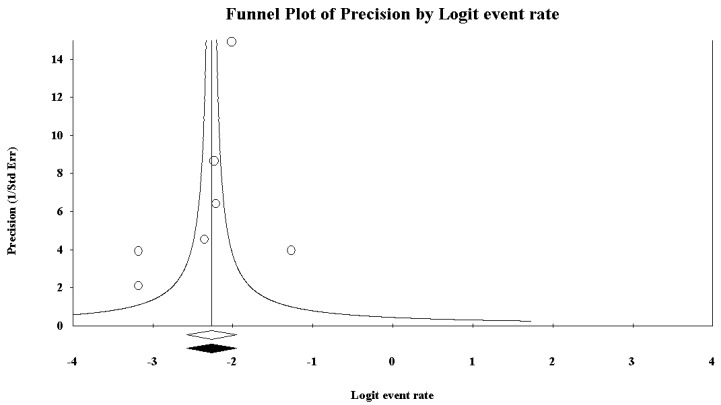
Funnel plot for “everyday drinking” consumption rate among truck-drivers, showing no evidence of publication bias.

Seven studies reported data concerning “everyday drinking” consumption rate among truck drivers (total population 4314 subjects, ranging from 91 to 2134 participants). Based on the *I*^2^ value (84.40), random-effects model was utilized. A rate of 9.4%, 95% CI (7.0, 12.4) was found (Figure 6). Sensitivity and cumulative analyses confirmed the stability of the findings. No statistically significant moderators were computed. Concerning the funnel plot (Figure 7), no evidence of publication bias could be detected, both visually inspecting the graph and performing the Duval and Tweedie’s trim-and-fill analysis and the Egger’s linear regression test (intercept = −1.98, standard error = 1.76, 95% CI (−6.51, 2.54), *t*-value = 1.13, *p* = 0.31111) (Table 7). 

### 3.4. Alcohol Consumption Rate among Truck Drivers Based on AUDIT-CAGE Instruments

**Figure 8:**
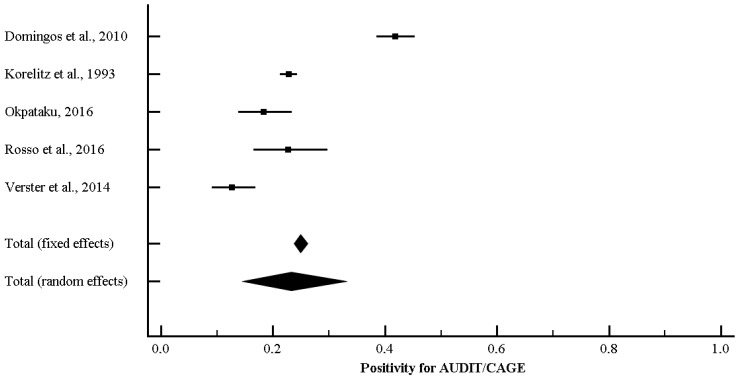
Forest plot for positivity to AUDIT-CAGE instruments rate among truck-drivers.

**Figure 9:**
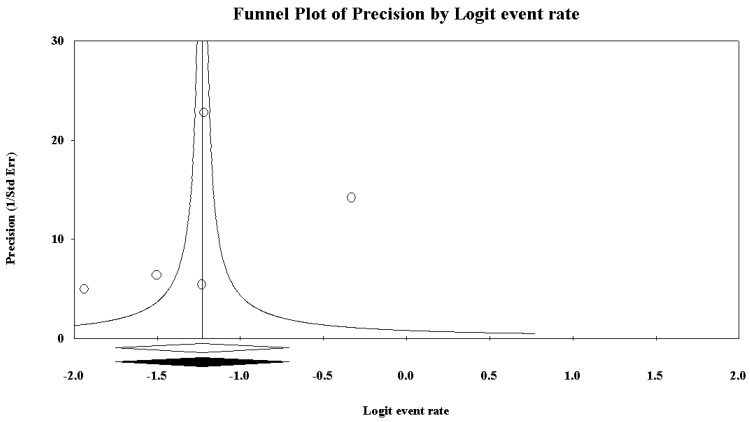
Funnel plot for alcohol consumption rate among truck-drivers based on the AUDIT-CAGE instruments, showing no evidence of publication bias.

Five studies reported data concerning alcohol consumption rate based on AUDIT-CAGE instruments. Based on the *I*^2^ value (97.29), random-effects model was carried out. A consumption rate of 22.7%, 95% CI (14.8, 33.0) was found (Figure 8). Concerning the funnel plot (Figure 9), no evidence of publication bias could be found, both visually inspecting the graph and carrying out the Duval and Tweedie’s trim-and-fill analysis and the Egger’s linear regression test (intercept = −1.84, standard error = 5.72, 95% CI (−20.04, 16.36), *t*-value = 0.32, *p* = 0.76889) (Table 8).

## 4. Discussion

The goal of this study was to assess the harmful alcohol consumption in the occupational category of truck drivers, a group of workers of the utmost importance for road safety and the global economy. In particular, we investigated three patterns of alcohol use considered hazardous or harmful: namely, “binge drinking”, “everyday drinking”, and positivity to AUDIT or CAGE tests.

The findings of the current meta-analysis showed a relevant harmful alcohol use prevalence among truck drivers. The results regarding “binge drinking” (prevalence of 19.0%) are extremely important because this mode of consumption has been linked to impairment in several executive cognitive functions [84,85] necessary for the complex task of driving. Frontal executive functioning of the brain is part of a system that controls the hierarchical order of brain processing, thus permitting control over cognition and behaviors [86,87]. Some authors have suggested that this executive impairment could impact on the predisposition towards the development of harmful habits including alcohol use disorders (AUD) [88,89] and use of illicit drugs. Moreover, this pattern has been associated with relevant road safety issues: “binge drinking” at least once a month increases the chance of being involved in crash accidents by ten-fold [90]. 

Interestingly, the results of the present study show a positive association between binge drinkers and nationality: the study showed that Brazilian drivers have a higher prevalence of “binge drinking” compared to their North American counterparts. This is comprehensible since alcohol consumption is, and has been, part of human culture, with different historical and social significance, as well as characterized by diverse patterns of consumption and different legislation in every country. Nevertheless, this observation needs further data: most studies that evaluated “binge drinking” were performed in Brazil, while only one was performed in the USA. 

Another association was found between “binge drinking” and marriage. Since most truck drivers spend significant amounts of time away from home, days and even weeks at a time, this result can be interpreted—as some authors have suggested—that being distant from families removes a valuable support system that acts as a barrier against stress [28].

Regarding the “everyday drinking” pattern, our results showed a prevalence of 9.4% in the studied population. This issue is particularly relevant for the specific occupational category of truck drivers, a group of workers that most likely drives every working day. Among drivers drinking everyday could be indicative of another alcohol use pattern: AUDs [57]. Indeed, “everyday drinking” puts the user on the threshold of at risk consumption even considering the AUDIT test.

Another important finding of the present study is the prevalence of positives to AUDIT-CAGE tests (22.7%). Although this significant prevalence, and despite the fact that the AUDIT instrument is a reliable and easy to use test, the real epidemiological figures could be even higher, in light of under-reporting during the Occupational Health Surveillance, as suggested by some authors [61].

Moreover, an association between testing positive and being overweight or obese has been reported in the literature [52]. It is well known that excess bodyweight is one of the major risk factors for OSA. Therefore it is not surprising that OSA is more prevalent in truck drivers than the general population [91]. Statistically significant rise in sleep apnea severity and cardiac frequency are induced by 0.5 g alcohol/kg body weight, a level regarded as the safe upper limit by health authorities, on sleep apnea in otherwise healthy habitual snorers with mild-to-moderate OSA [92]. Similar results were obtained in a truck driver sample [72,73]. 

Even if heterogeneity exists between countries worldwide, the results discussed above show various prevalence rates among truck drivers, approximately in line with the prevalence in the general population, as reported in Table 9. 

Many countries and international organizations have implemented policies aimed at reducing alcohol use among the general population. One of the guiding principles of the global strategy to reduce alcohol consumption adopted by the WHO is the “protection of populations at high risk of alcohol-attributable harm and those exposed to the effects of harmful drinking by others should be an integral part of policies addressing the harmful use of alcohol” [96]. The “European action plan to reduce the harmful use of alcohol 2012–2020” suggests a development of community and workplace resources for alcohol programs, and an enhanced enforcement of road alcohol tests as well as a reduction of the blood-alcohol content [97]. 

In Italy, the Prevention National Plan 2014–2018 [98] regards the prevention of substance use, the prevention of traffic accidents, and the prevention of occupational injuries and disorders as three of its ten macro-goals. It is thus clear that the issue is of importance both from a public health as well as from an occupational health perspective. However, there are several factors among truck drivers that must be accounted for: truck drivers are one of the occupational categories identified by the State-Regions Conference in 2006 as at high risk of injuries and harm to others, to which the sale and use of alcohol is prohibited. Furthermore, Italian driving law requires a total absence of blood alcohol concentration (0 g/L) for professional drivers (the so-called “zero effective tolerance” policy), while the threshold for the general population is 0.5 g/L. Not all countries have adopted such policy and, as such, the alcohol level at which a person is considered legally impaired differs among countries. 

A combined effect of a low quantity of alcohol with moderate sleep restriction results in significant decrements to subjective alertness and driving performance [99]. The detrimental effect of alcohol is also observed in relation to circadian sleep propensity. Even low consumption of alcohol could be too high when driving under a condition of increased sleep pressure such as during the night hours, in association to the effect of sleep deprivation. The highest values of hourly circadian sleep propensity are during the night, with a secondary maximum in the afternoon. Due to the significant interaction, even low BAC levels strongly increased road accident risk when associated with high sleep propensity [100].

Managing the issue of alcohol use among truck drivers is therefore bidirectional: public health policies can help reduce the consumption rate also among truck drivers, while occupational health prevention and health promotion will contribute to the reduction in the general population consumption. The first necessary step is to further study the epidemiology, especially in Europe, in order to better grasp the current state of the problem, with the aim of filling the current gap of knowledge supporting policy makers in implementing effective measures to contrast it.

## 5. Strengths and Weaknesses

To the best of the authors’ knowledge, this is the first systematic review and meta-analysis studying alcohol consumption in truck drivers. The strengths of this study include comprehensive coverage of the literature, careful appraisal of study quality, risk of bias, consideration of possible subgroup effects, and a focus on relevant endpoints to this specific occupational category. However, this study also presented a few shortcomings. 

One of the major limitations of this meta-analysis was the paucity of studies, especially from Europe. This finding is even more relevant if we consider that this region has the highest per capita alcohol consumption and alcohol-attributable disease burden reported in the world. Moreover, there is a high level of “binge drinking” among Europeans, as it is apparent from Table 9. There is a dearth of studies on truck drivers in European countries, for which no/little current epidemiological data are available, which means de facto ignoring the potential risk represented by harmful drinking pattern in this occupational category.

Such a geographical disparity might have influenced the results of the meta-analysis because of different socio-economic contexts and cultural backgrounds. Another important limitation in this study was the qualitative differences concerning the way of collecting data on alcohol use and the demographics of the population between studies.

Several studies had to be excluded from the meta-analysis because data on alcohol consumption in frequency/dose were missing, or because data were related to any consumption pattern in the past 12 months. Another limitation regarded the quantity and quality of the studies assessing the consumption during working hours using biological sampling: although seven studies evaluated alcohol content in bodily fluids, each one used different detection methods and cut-offs. In our assessment, only blood samples or breath correlates are indicative of recent alcohol use. Positive results in urine and saliva tests might be detected even after 48–72 h after alcohol consumption. Finally, as already mentioned in the material and methods section, caution should be taken in interpreting the findings of the statistical tests used for assessing the publication bias, given the high heterogeneity and the small number of included studies.

An improvement over these limitations is needed in order to better understand and evaluate the issue of alcohol use among truck drivers with a higher degree of accuracy. More studies using standardized questionnaires—thoroughly investigating the demographics, psycho-social determinants of the population, and well-defined patterns of alcohol use—need to be performed. Also, studies using comparable biological sampling methodologies are necessary.

## 6. Conclusions

This systematic review with meta-analysis provides the first rigorous analytical synthesis of updated epidemiological data regarding truck drivers. Our findings show that the prevalence of alcohol use among this occupational category can be considered harmful and put the light on some existing gaps, including the dearth of studies and data for many countries, but at the same time provide useful insights. This can be useful for decision and policy-makers in order to develop, design, and implement adequate surveillance and preventive policies. 

However, in order to better assess the magnitude of the risk, more specified and defined modes of consumption must be investigated.

## Figures and Tables

**Table 1:** Search strategies criteria of the current meta-analysis.

Search Strategy Item	Search Strategy
Databases	PubMed/MEDLINE (NLM), Scopus, SciVerse ScienceDirect, Science Citation Index Expanded and Social Sciences Citation Index from ISI/Web of Science, ProQuest Research Library, ABI/INFORM, CBCA, via the UNO per TUTTI Primo Central (Ex Libris) platform
Language filter	None
Time filter	None
Keywords	(truckers OR truck drivers OR lorry OR commercial vehicles OR large good vehicles OR large vehicles OR heavy vehicles OR long vehicles OR trucking industry OR haul transport) AND (alcohol OR ethanol)
Exclusion criteria	Editorial, letter to the editor, commentary, review; original article focusing on selected subgroups of truck drivers
Target journals	*Accident Analysis and Prevention*; *American Journal of Industrial Medicine*; *Applied Ergonomics*; *Ergonomics*; *Health and Place*; *Human Factors*; *International Archives of Occupational and Environmental Health*; *International Journal of Environmental Research and Public Health*; *International Journal of Heavy Vehicle Systems*; *International Journal of Industrial Ergonomics*; *Journal of Occupational and Environmental Medicine*; *Journal of Occupational Health*; *La Medicina del Lavoro/Medicine, Health and Working Life*; *Occupational and Environmental Medicine*; *Proceedings of the Human Factors and Ergonomics Society*; *Safety and Health at Work*; *Safety Science*; *Scandinavian Journal of Work, Environment and Health*; *Traffic Injury Prevention*; *Transportation Research Part F Traffic Psychology and Behavior*; *Transportation Research Record*; *Workplace Health and Safety*

**Table 2:** Data extracted from the included studies in the current meta-analysis.

Extracted Data	Details
Study Reference	Names and surnames of authors, year of publication
Country	Country or countries in which the study or studies was or were carried out
Study design	Type of recruitment
Male % (M%)	Percentage of male truck drivers
Age	Mean age of the truck drivers sample
Sample number, attrition rate	Number of truck drivers, number of non-responders
Marital status	Married or in a union; single, separated, divorced, widowed; with or without children
Schooling level	Maximum educational level attained by the truck driver
Religious practice	Whether the truck driver is religious (for example, Christian, Jewish or Muslim) or not
Professional years	Years spent in profession by truck drivers included in the study
Work load	Expressed in hours
Monthly income	Average earning
Mean distance (km)	Distance travelled in the last shipment
Duration of the trip	Duration expressed in days
Interstate destination	Whether the destination of the truck driver is interstate or not
Co-morbidities prevalence (%)	Health problems suffered from truck drivers included in the study
Truck ownership	If the driver or the employer owns the truck
Working for companies (%)	Whether the truck driver works for a company or not
Period of the day driving the most	Day, day and night, night shift
If the truck is tracked by satellite	Solo drivers (%)
Ethnicity	Nationalities of drivers included in the study
Having another job	Whether the truck driver has a further job and which one
Patterns of alcohol use (prevalence rate)	Different pattern rates of alcohol use (binge drinking, positivity to AUDIT/CAGE tests, “everyday drinking”)
Method utilized to investigate patterns of alcohol use	Questionnaire (validated, not validated); urine samples, blood samples, breath samples, saliva

Abbreviations: AUDIT (Alcohol Use Disorders Identification Test); CAGE (Cut down-Annoyed-Guilty-Eye opener questionnaire).

**Table 3:** Characteristics of the studies excluded with reason from the meta-analysis for methodological heterogeneity related to the definition of alcohol consumption pattern.

Study	Sample Size	Consumption Rate (%)	Alcohol Consumption Definition
Questionnaire-based			
De Oliveira et al., 2015 [68]	514	0.77	Generic consumption rate during the last year
Gay Anderson et al., 2008 [28]	987	0.63	Generic consumption rate during the last year
Lemire et al., 2002 [69]	2167	0.61 ≤ 2 drinks/week, 0.26 3–6 drinks/week, 0.02 > 15 drinks/week; 0.01 admitted to drink on the assessment day	Number of drinks/week
Mansur Ade et al., 2015 [70]	2228	0.23–0.37	Non-specified alcohol consumption
Maarefvand et al., 2016 [71]	349	0.014	Non-specified alcohol consumption
Masson and Monteiro, 2010 [72,73]	105	0.495	Non-specified alcohol consumption
Riva et al., 2010 [74]	226	0.51 non-usual drinkers, 0.47 < 0.5 L alcohol/day 0.03 > 0.5 L alcohol/day	Consumption in terms of L alcohol/day
Sakurai et al., 2007 [75]	1465	0.25 < 0.5 g alcohol/kg; 0.22 0.5–1 g alcohol/kg; 0.07 > 1 g alcohol/kg	Consumption in terms of g alcohol/kg
Sangaleti et al., 2014 [76]	250	0.668	Non-specified alcohol consumption
Takitane et al., 2013 [77]	130	0.692	Non-specified alcohol consumption
Yonamine et al., 2013 [78]	1277	0.259	Non-specified alcohol consumption
Biological monitoring—saliva			
Gjerde et al., 2012 [79]	882	0.01	Automated enzymatic method using alcohol dehydrogenase (cut-off 0.2 g/L)
Yonamine et al., 2013 [78]	1250	0.01	Headspace-gas chromatography-flame ionization detection method (cut-off 0.2 g/L)
Biological monitoring—urine			
Couper et al., 2002 [80]	822	0.013	Headspace-gas chromatography-flame ionization detection method (GCFID)
Labat 2008 [81]	1000	0.05	Enzymatic technique was used for ethanol determination. Detection limit was estimated at 0.1 g/L
Biological monitoring—breath			
Drummer et al., 2007 [38,39]	3974	0.01	Breath test 0.5 g/L
Woratanarat et al., 2009 [82]	200	0.05	Breath test (Lion Alco meter SD-400)
Biological monitoring—blood			
Lund et al., 1988 [83]	299	0.003	Gas chromatography with a nominal detection threshold of 0.01 g/dL in blood or urine, three blood positives with values of 0.01,0.02 and 0.03 g/dL

**Table 4:** Characteristics of the studies included in the current meta-analysis.

Study	Sample Size	Binge Drinking	AUDIT/CAGE	Daily Drinking	Country	Age	Male	Marriage	Mean Distance	Experience Years	Work Load	For Companies	Schooling Level	Night Shift
De Oliveira et al., 2016 [12]	391	0.175	NR	NR	Brazil	37.7	NR	75.72	1149	12.41	11.99	57.03	NR	20.72
Domingos et al., 2010 [55]	827	NR	0.418	NR	Brazil	41.3	99.3	85.5	NR	NR	NR	NR	67	NR
Girotto et al., 2015 [27]	670	0.291	NR	NR	Brazil	41.9	100	NR	934.1	18.1	NR	NR	58.2	NR
Jora et al., 2010 [56]	496	0.258	NR	NR	Brazil	41.8	95.2	79	NR	NR	NR	NR	NR	NR
Knauth et al., 2011 [57]	854	NR	NR	0.097	Brazil	NR	100	83.8	NR	NR	NR	NR	30.8	NR
Korelitz et al., 1993 [26]	2945	NR	0.228	NR	USA	NR	89	69.6	NR	NR	NR	NR	81	NR
Laraqui et al., 2011 [58]	2134	NR	NR	0.118	Morocco	NR	100	NR	NR	12.2	11.1	NR	NR	19.4
Leopoldo et al., 2015 [59]	535	0.174	NR	NR	Brazil	37.8	100	74.7	1127.3	12.5	12.1	60.9	48.8	12.5
Mir et al., 2012 [8]	461	NR	NR	0.099	Pakistan	NR	NR	NR	NR	NR	NR	NR	NR	NR
Nascimento et al., 2007 [60]	91	NR	NR	0.22	Brazil	NR	100	NR	NR	10	NR	NR	NR	33
Okpataku, 2016 [61]	274	NR	0.182	NR	Nigeria	43.4	100	94.9	NR	NR	NR	NR	67.5	NR
Penteado et al., 2008 [62]	400	NR	NR	0.04	Brazil	42.2	NR	NR	NR	NR	12.7	40.5	NR	NR
Pinheiro et al., 2015 [63]	114	NR	NR	0.04	Brazil	NR	100	62	NR	NR	NR	NR	38	NR
Rosso et al., 2016 [64]	168	NR	0.226	NR	Italy	42.7	NR	NR	NR	18	NR	NR	65	NR
Souza et al., 2005 [65]	260	NR	NR	0.087	Brazil	38.2	100	76.6	NR	NR	NR	NR	71.3	NR
Valway et al., 2009 [66]	652	0.0987	NR	NR	USA	44	90.6	51.7	NR	13	NR	76	78.5	NR
Verster et al., 2014 [67]	302	NR	0.126	NR	The Netherlands	33.8	95.6	NR	NR	12.6	NR	NR	NR	NR

**Table 5:** Quality assessment of the studies included in the current meta-analysis.

Study	Domain i	Domain ii	Domain iii	Domain iv	Domain v	Domain vi	Domain vii	Domain viii	Domain ix
De Oliveira et al., 2016 [12]	Yes	No	Yes	Yes	Yes	Yes	Yes	Yes	Yes
Domingos et al., 2010 [55]	Yes	No	Yes	Yes	Yes	Yes	Yes	Yes	Yes
Girotto et al., 2015 [27]	Yes	No	Yes	Yes	Yes	Yes	Yes	Yes	Yes
Jora et al., 2010 [56]	Yes	No	Yes	Yes	Yes	No	No	Yes	Yes
Knauth et al., 2011 [57]	Yes	No	Yes	No	Yes	No	No	Yes	Yes
Korelitz et al., 1993 [26]	Yes	No	Yes	No	Yes	Yes	Yes	Yes	Yes
Laraqui et al., 2011 [58]	Yes	No	Yes	Yes	Yes	No	no	Yes	Yes
Leopoldo et al., 2015 [59]	Yes	No	Yes	Yes	Yes	Yes	Yes	Yes	Yes
Mir et al., 2012 [8]	Yes	Yes	Yes	No	Yes	No	No	Yes	Yes
Nascimento et al., 2007 [60]	Yes	No	No	No	Yes	No	No	Yes	Yes
Okpataku, 2016 [61]	Yes	Yes	Yes	Yes	Yes	Yes	Yes	Yes	Yes
Penteado et al., 2008 [62]	Yes	No	Yes	Yes	Yes	No	No	Yes	Yes
Pinheiro et al., 2015 [63]	Yes	No	No	Yes	Yes	No	No	Yes	Yes
Rosso et al., 2016 [64]	Yes	No	Yes	No	Yes	Yes	Yes	Yes	Yes
Souza et al., 2005 [65]	Yes	No	Yes	Yes	Yes	No	No	Yes	Yes
Valway et al., 2009 [66]	Yes	No	Yes	Yes	Yes	Yes	Yes	Yes	Yes
Verster et al., 2014 [67]	Yes	No	Yes	No	Yes	Yes	Yes	Yes	Yes

Domain i concerns the appropriateness of the sample frame to address the target population; Domain ii, the participants sampling technique; Domain iii, the adequateness of the sample size; Domain iv, the completeness of the description and details concerning the study subjects and the setting; Domain v, the coverage of the sample; Domain vi, the validity of the methods and Domain vii, their reliability; Domain viii, the appropriateness of the statistical analyses; and Domain ix, the adequateness of the response rate.

**Table 6:** Duval and Tweedie’s trim-and-fill analysis for binge drinking rate among truck drivers.

		Random-Effects Model			*Q* Value
	Studies Trimmed	Point Estimate	Lower Limit	Upper Limit	
Observed values		0.19	0.13	0.27	85.55
Adjusted values	0	0.19	0.13	0.27	85.55

**Table 7:** Duval and Tweedie’s trim-and-fill analysis for “everyday drinking” consumption rate among truck drivers.

		Random-Effects Model			*Q* Value
	Studies Trimmed	Point Estimate	Lower Limit	Upper Limit	
Observed values		0.09	0.07	0.12	38.45
Adjusted values	0	0.09	0.07	0.12	38.45

**Table 8:** Duval and Tweedie’s trim-and-fill analysis for alcohol consumption rate among truck-drivers based on the AUDIT-CAGE instruments.

		Random-Effects Model			*Q* Value
	Studies Trimmed	Point Estimate	Lower Limit	Upper Limit	
Observed values		0.23	0.15	0.33	147.59
Adjusted values	0	0.23	0.15	0.33	147.59

**Table 9:** Alcohol consumption in the general population 15+ years old.

Country	Harmful Consumption Rate (Risk Drinking, Heavy Episodic Drinking)	Consumption Rate in the Past 12 Months
EU27 2010 (Eurobarometer) [93]	32.7%, past month	76%
Italy 2016 (National Institute of Statistics or ISTAT) [94]	15.9%, past year	64.7%
Brazil 2010 (WHO), male [9]	20.7%, past month	69.3%
USA 2015 (National Survey on Drug Use and Health or NSUDH) [95]	26.9%, past month	70.1%
The Netherlands 2010 (WHO), male [9]	10.5%, past month	92.9%
